# The Influence of a Multimodal Cognitive Behavioural Intervention on the Stress Mindset, Psychological Wellbeing, and Performance of Students Aged 16–18 Facing Exams

**DOI:** 10.1002/smi.70075

**Published:** 2025-07-14

**Authors:** Katherine V. Sparks, Paul Mansell, Jason Wright, Matthew Slater

**Affiliations:** ^1^ University of Staffordshire Stoke‐on‐Trent UK

**Keywords:** academic performance, mental wellbeing, multimodal intervention, rational emotive behavioural therapy, self‐compassion, stress mindset

## Abstract

Exam stress is one of the most influential factors for adolescent students' mental wellbeing (Brown et al. 2022). The typical response is to try and avoid, reduce or eliminate the stress, but it is possible to change the way individuals appraise stress. The present study aimed to investigate the effect of a 6‐week multi‐modal intervention on markers of performance and mental wellbeing in students that were soon to be completing their secondary and tertiary examination period (16–18 years). The intervention employed a multi‐modal approach of stress psychoeducation, self‐compassion and reappraisal of irrational beliefs. Content included helpful thinking techniques, badness scale and imagery to promote a more beneficial psychological response to exams. Eighty‐six young persons (49 males and 37 females) aged between 16 and 18 years old (*M* = 16.92, SD = 0.99) participated, 55 participants in the experimental group and 31 participants in the control group. All participants completed measures of stress‐mindset, perceived performance, irrational beliefs, anxiety, depression and proactive coping at baseline and post‐intervention. The separate ANCOVA's revealed there was greater significant levels of stress‐mindset, perceived performance and proactive coping in the experimental compared to the control at post. Furthermore, there was significantly lower levels of depression in the experimental compared to the control from at post. No significant changes were found in irrational beliefs and anxiety at post. Overall, the multi‐modal approach demonstrates to be efficacious in aiding a young person's mental wellbeing and performance.

## 1 Introduction

The dominant cultural perspective of stress equates to distress; therefore, we must try to avoid, reduce, or eliminate it (Brooks 2014). This may be unsurprising with several studies demonstrating high levels of perceived stress among adolescent students are linked to concerning factors such as poor mental wellbeing (Brown et al. 2022). In fact, the secondary and tertiary examination period is one of the most influential factors for adolescent students (16–18 years) on their mental wellbeing due to the examination stress (Brown et al. 2022). The link between exam stress and mental health difficulties is ever growing stronger (Brown et al. 2022; Roome and Soan 2019) and a prominent association between a young person's wellbeing and their future wellbeing, therefore it is essential to explore ways to help students manage the demands of exams (Aldridge and McChesney 2018). In the present research, we aim to work with adolescents to develop their mindset—their general assumptions and beliefs about something (e.g., the stress of exams)—to lead to more sustainable and desirable outcomes (Crum et al. 2023).

### 1.1 Wellbeing, Stress Mindsets, and Performance

Wellbeing is multifaceted and encompasses physical, social, and psychological components (Jarden and Roache 2023). In the current study, we have focussed on aiding psychological wellbeing. There is no clear universally agreed conceptualisation of wellbeing (Jarden and Roache 2023), various differing frameworks exist (Bradburn 1969; Ryan and Deci 2000; Ryff and Singer 1998; Diener 1984; Waterman 1993), and there are multiple scales (Rose et al. 2017). There are two relatively distinct, yet overlapping, perspectives of wellbeing: (1) Hedonism (Kahneman et al. 1999), consisting of pleasure versus displeasure and happiness; and (2) Eudaimonism (Waterman 1993), consisting of fulfilment and actualisation, living in accordance with one's true self. Wellbeing frameworks tend to lean on one perspective over the other. For instance, Diener's (1984) introduced the concept of subjective psychological wellbeing, a tripartite framework that takes the hedonism perspective of wellbeing, emphasising that positive mental states are not merely the absence of distress but the presence of frequent experiences of positive affect (infrequent negative affect) and life satisfaction as close to ideal as possible. While subjective psychological wellbeing is a well‐established construct in wellbeing literature, there is considerable debate about whether it sufficiently captures the full scope of psychological wellness (Ryff and Singer 1998). Ryff and Singer's (1998) framework of wellbeing takes the eudaimonism perspective, focussing on psychological functioning and self‐actualisation, through six dimensions: autonomy, self‐acceptance, purpose in life, mastery, positive relatedness, and personal growth. Here, there are similarities with self‐determination theory, within which it is proposed that the fulfilment of the three psychological needs—autonomy, competence and relatedness—is crucial for psychological growth, personal integrity, and overall wellbeing (Ryan and Deci 2000). However, Keyes (1998) argued that despite being an integral facet of subjective psychological wellbeing, the social aspect of wellbeing is not adequately captured within these frameworks. Individuals are inherently embedded within social structures and communities, therefore our social functioning and the way we relate to others both influence our social wellness and overall psychological functioning. Collectively, there is considerable overlap between the hedonism and eudaimonism wellbeing (Thorsteinsen and Vittersø 2020), consequently in the current research we assessed psychological wellbeing using measures of anxiety, depression, and proactive coping.

It is well established that stressful life events and maladaptive coping are linked to poor psychological wellbeing (Zhou et al. 2023). Adolescents coin examinations as a stressful life event and this appraisal can have a significant influence on their psychological wellbeing (Högberg 2021; Zhou et al. 2023). Consequently, individuals' perception of stress may play an integral role in influencing wellbeing, which offers an opportunity for intervention (Huebschmann and Sheets 2020; P. C. Mansell and Turner 2023).

Crum and colleagues (2013) proposed that there are two types of mindsets towards stress; stress‐is‐enhancing or stress‐is‐debilitating. A stress‐is‐enhancing mindset is where an individual believes that stress can be beneficial and results in positive outcomes including performance, productivity, and wellbeing. A stress‐is‐debilitating mindset is the opposite in that stress is detrimental and can have negative outcomes (Keech et al. 2020). In relation to academic performance and student wellbeing, Wang et al. (2022) demonstrated that a stress‐is‐enhancing mindset towards upcoming exams is linked to challenge appraisals, while stress‐is‐debilitating mindset is linked to threat appraisals.

Within the Theory of Challenge and Threat States (TCTSA‐R; Meijen et al. 2020), it is proposed that an individual will make an initial appraisal of a situation as a challenge or threat depending on the situation importance (e.g., ‘a goal at stake or not’) and the goal congruence (e.g., ‘situation is favourable for success or not’). For instance, if the student perceives the importance of the exam and goal congruence high, they may perceive the situation as a challenge (likely to be successful), opposite would be true for a threat appraisal. Following this, they will appraise whether their resources (self‐efficacy, perceived controllability, and type of goal focus) meet the demands of the situation or not. Students who appraise that their resources meet or outweigh the demands, would have high self‐efficacy, perceived controllability, and approach goal focus. In contrast, a student who appraises that the demands outweigh their resources would have low self‐efficacy, perceived controllability and exhibit an avoidance approach focus (Jones et al. 2009). Depending on their perception of their resources to the demands, this will either reduce or reinforce their initial appraisal of challenge or threat. Moreover, threat compared to challenge appraisals to stressful situations are associated with poor mental health, namely depression and anxiety symptoms (McLoughlin et al. 2024).

Challenge and threat evaluations may also play a mediating role. Wang et al. (2022) also found that a stress‐is‐enhancing mindset had an indirect positive effect on their exam performance when mediated by challenge rather than threat appraisals. This suggests that students who perceive stress as beneficial appraised exams as a challenge and had a better exam performance than those who perceived exam stress as detrimental and entered a threat state. In addition, a stress‐is‐enhancing mindset was linked to lower anxiety, stress, and depression levels suggesting that when individuals acknowledge the good side of stress, this can in turn reduce our perceived stress levels. Although this provides promising initial evidence that altering students' stress mindset may be beneficial, applied research that examines this proposition via evidence‐based interventions has not been forthcoming. In addition, there is a lack of research in adolescent students exploring both academic performance and wellbeing.

Similarly, stress mindsets have been linked to adaptative coping skills in young adults which are pivotal for dealing with stressful situations effectively and protecting mental wellbeing (Chen et al. 2022). Chen et al. (2022) found stress‐is‐enhancing compared to stress‐is‐debilitating mindsets were related to greater coping flexibility in undergraduate students. Therefore, those with a stress‐is‐enhancing mindset had the ability to adaptatively monitor and modify their coping strategies to meet situational demands. Furthermore, a student's stress‐mindset and coping flexibility mediated the relationship between recent college stressful experiences (e.g., meeting academic standards) and psychological distress. In other words, students who exhibited a stress‐is‐enhancing mindset had greater coping flexibility and this in turn decreased their psychological distress in response to college stressors, protecting their mental wellbeing compared to those with a stress‐is‐debilitating mindset. Although conducted in university students rather than adolescents, these findings show the potential influence of the stress‐mindset on coping skills and wellbeing markers, therefore warranting investigation.

### 1.2 Changing Stress Mindsets and Irrational Beliefs

Individuals with stress‐is‐debilitating mindsets consider stress as only bad, and this has been posited to be akin to being an irrational belief about stress (P. C. Mansell 2021). Compared to rational beliefs which are flexible, truthful, and logical, irrational beliefs are rigid, extreme, and illogical. They comprise of a primary irrational belief demandingness that is defined by absolutes (e.g., ‘I must’), which may be coupled with three secondary irrational beliefs of low frustration tolerance, self‐depreciation, and awfulizing (Ellis and Dryden 1997). According to the ABC(DE) framework, the theoretical underpinning of the Rational Emotive Behavioural Therapy (REBT, Ellis and Dryden 1997), is that individuals tend to adopt A to C thinking, whereby an Activating (A) event results in behavioural and emotional Consequences (C) leading to lack of control and problematic thinking. Yet A to C thinking misses the crucial role of an individual's Beliefs (B). In other words, it is not the activating event (A) that causes the consequences (C) but the beliefs (B) that an individual has about the event. Depending on the type of belief (irrational or rational) an individual holds will determine whether they experience a positive or negative consequences such as thoughts, emotions, and behaviours (Szentagotai and Jones 2010). In terms of stress, an individual exposed to an acute stress (A), who believes that all stress is bad (B) will respond in a dysfunctional manner (C) such as avoiding any situation that elicits stress, which is not adaptable or always feasible (e.g., in exams). Irrational beliefs, more generally in secondary school students have been linked to a greater increase in exam‐related anxiety towards the testing period and are related to dysfunctional distress such as clinical anxiety and depression (Dilorenzo et al. 2011). In contrast, rational beliefs have been found to reduce the level of exam‐related anxiety, leading to a decrease in dysfunctional but an increase in functional distress (i.e., feelings of concern and sadness; normal negative responses to stressful events; Dilorenzo et al. 2011).

Through REBT an individual's irrational belief is Disputed (D) and replaced with a rational belief leading to more functional emotions and Effective (E) behaviour. REBT is a robust framework used to inform interventions to reduce irrational beliefs and aid psychological wellbeing (M. J. Turner 2016). It is well‐established within the adolescent population that REBT informed interventions are effective in changing beliefs (Eifediyi et al. 2018; Sari et al. 2022; Mosimege et al. 2024). Specifically, Mosimege et al. (2024) reported that secondary school students who participated in group REBT sessions had significant reductions in irrational beliefs, tension, and anxiety regarding mathematics compared to a control group. Overall, such evidence provides promise in the implementation of an REBT informed intervention, but studies have been mainly conducted in students outside the United Kingdon and not examined ahead of pivotal examinations. This, therefore, requires investigation and is addressed in the current study. Furthermore, specific to stress, limited research has been conducted between REBT and stress mindsets. Despite this, psychoeducation in relation to reframing stress, an REBT disputation technique, has been implemented through video or instruction, which has demonstrated to adaptatively change an individual's stress‐mindset (Crum et al. 2017).

In Australian college students, Keech, Hagger, et al. 2021 conducted a brief intervention whereby the experimental group were given a combination of stress education and imagery, which included a balanced overview of stress and how student‐related stressors could have positive consequences. Results indicated that students in the intervention had a greater stress‐is‐enhancing mindset compared to baseline and the control condition. Moreover, students with high baseline levels of perceived distress, following the intervention, had lower distress levels, improved coping skills, and academic performance. Nevertheless, there was no immediate upcoming stressor therefore all self‐report assessments were retrospective. Generally, these brief interventions have demonstrated some success, however, to have a sustained impact it is important to target what underpins an individual's stress mindset, such as irrational beliefs. Therefore, an intervention helping students facing exams underpinned by REBT may be a fruitful approach in reducing irrational beliefs and enhancing stress‐related outcomes.

### 1.3 Self‐Compassion and Mental Wellbeing

Unconditional self‐acceptance is one of the main assumptions of REBT (Ellis and Dryden 1997) and it is conceptualised as the tendency to fully accept oneself no matter the outcome (Ellis 2005). Self‐compassion cultivates this through the acceptance of oneself in terms of fallibility and absence of self‐judgement (Mosewich 2020).

Self‐compassion is formed of three elements: self‐kindness, mindful awareness, and common humanity (K. D. Neff 2023). Self‐kindness refers to the tendency to be caring and understanding to oneself. Mindfulness is being aware of the present moment experience and not ruminating the past or pre‐empting the future. Lastly, common humanity is recognising that we are all fallible human beings, we are not alone in imperfection or difficulty. There is growing evidence in the beneficial role that self‐compassion can have on an adolescent's mental health (Marsh et al. 2018), including reducing levels of anxiety, depression and stress (Marsh et al. 2018) and using less maladaptive coping strategies (Ewert et al. 2021).

In relation to examinations which could be coined as a high‐stake situation due to failure being possible, this can increase an individual's stress levels (Ceccarelli et al. 2019). Recent research has demonstrated that adolescents with higher levels of self‐compassion exhibited lower levels of test anxiety, suggesting the protective role that self‐compassion could play (O’Driscoll and McAleese 2023). Furthermore, self‐compassion is associated with more adaptable thoughts about failure, therefore may reduce the irrational thoughts that may surround success and failure, as these can further exacerbate perceived stress towards an examination (Chan and Sun 2021). For instance, Stephenson et al. (2018) found self‐compassion was negatively related to irrational beliefs such as low frustration tolerance. Similarly, self‐compassion has been found to work as a buffer to irrational beliefs, and in turn, reduce the likelihood of depression (Podina et al. 2015). Therefore, given the evidence presented, integrating self‐compassion within this type of intervention for students facing exam could be impactful.

### 1.4 Multi‐Model Interventions

Overall, stress mindset interventions have adopted predominately a unimodal approach, with the majority using educational videos to reduce the global negative perception of stress and decrease irrational beliefs (e.g., Crum et al. 2017). Similar, school‐based interventions have taken a similar approach, using one therapeutic approach, namely mindfulness (Zenner et al. 2014; Zhou et al. 2020). Nevertheless, a multimodal approach may prove to be more efficacious, for instance, in a sister study in young athletes, utilising the same multi‐modal intervention as the current study; a combination of stress psychoeducation, awareness of the ABC thinking framework, self‐compassion and imagery resulted in increases in stress mindset, perceived performance, and reductions in negative affect (P. Mansell et al. 2023).

### 1.5 Current Study

In this study we apply a multi modal cognitive approach encompassing both REBT and self‐compassion (P. Mansell et al. 2023) to enhance students' stress‐mindset, reduce irrational beliefs, improve mental wellbeing, and increase performance in the context of upcoming exams. Against the backdrop presented, we hypothesise that there will be greater post stress‐mindset scores in the experimental group, compared to the control group (H1). Additionally, we expect that there will be a significantly differences in wellbeing markers post intervention, namely lower irrational beliefs, anxiety and depression but greater proactive coping scores in the experimental group compared to the control group (H2). Similarly, we hypothesise there will be a significantly higher post scores in perceived academic performance in the experimental compared to the control group (H3).

## 2 Method

### 2.1 Participants and Design

Following the approval from the institution's ethics committee, we recruited 86 students (49 males and 37 females) aged between 16 and 18 years (*M* = 16.92, SD = 0.99) through convenience sampling in one UK state secondary school, two colleges, and one private school. All participants were required to be sitting their General Certificate of Secondary Education (GCSE) or Advanced level (A LEVEL) exams in the upcoming summer exam period, and to be proficient in reading English. There was no exclusion criteria as the study were to be as inclusive as possible. A total of 14 participants dropped out within the 6 weeks and did not complete the post questionnaire (*n* = 11 experimental, and *n* = 3 control). Overall, adopting a 2 (condition: experimental and control) X 2 (time: baseline vs. post intervention) design there were 44 students in the experimental group (26 males, 18 females) with a *M*age of 16.68 (SD = 0.96) and 28 students in the control group (17 males, 11 females) with a *M*age = 17.57 (SD = 0.57). An apriori power calculation was completed on GPower (Faul et al. 2007), based on a power of 0.80 and an alpha of 0.05. Established by comparable study that implemented a 2 × 2 design and aimed to influence the same primary variable stress mindset they reported a large effect size (Keech, Hagger, et al. 2021), 24 participants per condition would provide sufficient power in our study. However, to allow for a 10% attrition rate we aimed to recruit at least 27 participants within each group.

### 2.2 Procedure

Senior leadership personnel who were overseeing GCSE and A level provision at the institutes were contacted with the opportunity to register their interest in the project. Senior leadership personnel were then asked to advertise the research to all students who had upcoming assessments (GCSE and A levels). This was achieved through assembly, form time announcements and/or emails. Students then had to voluntarily express interest to the school senior leadership personal, whereby an information sheet for the study was then sent by email to parents/guardians and students. Students and parents who still showed an interest were then requested to provide consent. Parental consent had to be provided before the first session, following this, students were then given the time to provide informed consent at the start of session one for those within the experimental group. Students who did not show an interest for the workshops were provided the opportunity to be in a control group and to use the time for revision. A similar process was followed, information sheets and informed consent were sent home to parents/guardians and students to complete before the first extra revision session. To maintain a manageable class size, a maximum of 15 students were allocated to a session, if this has been exceeded then repeats of the sessions would be completed. The same was considered for the control group with the aim of equal groups. However, half of the schools did not have enough uptake of participants to fairly divide the groups and therefore only provided an experimental group.

### 2.3 Measures

#### 2.3.1 Stress Mindset

Stress Control Mindset Measure (SCMM; Keech, Orbell, et al. 2021) was used to evaluate the stress mindset of individuals. The scale included 15‐items which was divided into 4‐subscales that covered stress beliefs about health and vitality (*‘Stress can be used to enhance your health and vitality’*), performance and productivity (*‘Stress can be used to enhance your performance and productivity’*), learning and growth (*‘Stress can be used to enhance your learning and growth’*) and in the general domain (*‘The effect of stress on you is negative’*). The response format was a six‐point Likert scale, whereby participants were asked to indicate how much they agreed with each of the statements from 1 (*strongly disagree*) to 6 (*strongly agree*). Negatively worded items were reverse scored and then all items were averaged together so that a higher value represented a more ‘stress‐is‐enhancing’ mindset. The SCMM has previously demonstrated good validity and reliability for measuring stress‐mindset (Keech, Orbell, et al. 2021). The scale revealed Cronbach alpha coefficient of 0.68 indicating a fair level of internal reliability.

#### 2.3.2 Irrational Beliefs

The Irrational Performance Beliefs Inventory (iPBI; M. J. Turner et al. 2016) is a 28‐item self‐report measure used to assess irrational beliefs. It is formed of four subscales including Demandingness (DEM), Low Frustration Tolerance (LFT), Awfulizing (AWF) and Depreciation (DEP). An example item is ‘*Decisions that affect me must be justified*’ which captures Demandingness. Responses are made on a five point Likert scale, and respondents are asked to rate their agreeableness with each of the statements from 1 (*strongly disagree*) to 5 (*totally agree*). In this study all 28‐items were summed to provide a total score of irrational beliefs, rather than dividing them into their subscale scores. The has previously demonstrated good criterion, construct and concurrent validity and reliability (M. J. Turner et al. 2018). The Cronbach alpha coefficient was 0.93 indicating excellent levels of internal reliability.

#### 2.3.3 Anxiety and Depression

The 14‐item Hospital Anxiety and Depression Scale assessed individuals' levels of depression and anxiety (HADS; Zigmond and Snaith 1983). The scale is divided equally into two subscales, measuring trait anxiety (e.g., *‘I get sudden feelings of panic’*) and depression (e.g. ‘I still enjoy the things I used to enjoy’). Respondents identify how they have been feeling over the past 2 weeks on a four‐point Likert scale ranging from 0 to three (e.g. not at all to most of the time). Several of the items are reverse scored before all items are summed with a higher score indicating a higher trait anxiety and depression. The HADS has demonstrated adequate validity and reliability within the adolescent population in screening for depression and anxiety (White et al. 1999). In the present study, the Cronbach alpha coefficient for anxiety was 0.84 and depression was 0.68, indicating a very good and fair reliability score respectively.

### 2.4 Proactive Coping

The 14 items proactive coping scale was a subscale from the multidimensional Proactive Coping Inventory (Greenglass et al. 1999). Participants rated each statement (e.g., ‘*I turn obstacles into positive experiences*’) on a four‐point Likert scale ranging from 1 (*not at all true*) to 4 (*completely true*). Negative worded items were reverse scored, following this all items were summed to create a total mean score. Higher scores indicated a greater proactive coping tendency. In the current study, the Cronbach alpha coefficient was 0.76.

### 2.5 Perceived Performance

Participants were asked to rate their academic performance over the last 2 weeks between 0% (extremely poorly) and 100% (extremely well), similar approach has been used previously for measuring athletic performance (e.g. M. J. Turner et al. 2021).

### 2.6 Manipulation Check

Two single‐item measures were used to assess whether the participant has engaged with intervention content. Using seven‐point Likert scale participants rated from 1 (*not at all*) to 7 (*very much so*) how much they engaged with the imagery content in session 6 and the wider intervention activities.

### 2.7 Stress‐Mindset for Under Pressure Intervention

We adapted and applied the same intervention implemented in P. Mansell et al. (2023) for the exam context (Supplementary Information S1: Appendix). The intervention consisted of 6 × 1 h weekly group‐based sessions and was underpinned by the REBT framework and included elements of self‐compassion. Below, we outline the intervention and adaptations made for the exam context.

#### 2.7.1 Week One—Overview of Intervention and Introduction to Stress‐Mindset

This covered an overview of what the intervention and participant expectations. Participants explored their current views of stress and their stress mindset. They were then introduced to the stress mindset and the paradoxical view of stress (e.g., using an educational video outlining the balanced perspective of stress, presenting the positive physiological stress responses but also the negative impact of chronic stress). Homework was then set to explain the stress mindset to a friend, family member or teacher.

#### 2.7.2 Week Two—Application of Stress Mindset and Challenge and Threat

Participants learnt about the three‐step approach to a stressful situation through a video outlining how to acknowledge the stress, appraise the stress helpfully, and harness their stress responses. They then completed the controlling the controllables task in preparation to their exams to help facilitate a challenge state. Homework included the participants to repeat the control mapping task to another pressured event for example sport fixture.

#### 2.7.3 Week Three—ABC Framework and Beliefs Towards Exams

We introduced the ABC framework and applied this to the upcoming exams. Students learnt about the difference between rational and irrational beliefs in the context of their exams and explored their own. Following this they completed the Badness Scale in small groups in relation to 10 possible adversities they may face at school or outside of school (M. Turner and Barker 2013). Following this, homework was set to outline 3–5 helpful belief statements they could have towards their exams.

#### 2.7.4 Week Four—Using Self‐Compassion for Helpful Thinking

Utilising the three elements of self‐compassion, students learnt to acknowledge, share and show self‐kindness to their thoughts and feelings towards exams (e.g., students identified their common thought towards exams and posted it on the ‘fear wall’, this presented mindfulness, a sense of acknowledgement of how they thought, and common humanity as students revealed similar disruptive thoughts). Following this, they were asked to be their own support coach and provide an alternative self‐kind statement they could say towards their exams. They were then asked to practice the support coach strategy when unhelpful thoughts arise for homework.

#### 2.7.5 Week Five—Understanding Imagery and its Benefits for Performance

Students learnt what imagery is and experienced an imagery script for themselves which was based around the three‐step approach as taught in Week 2. Following, this they wrote their own using the three‐step prompts. In terms of their homework, they were required to record and listen to their imagery script.

#### 2.7.6 Week Six—Recap of the Intervention

The final session included an overview of the intervention which was tailored to the group depending on what they wanted to re‐cover and an action plan to how they would implement their learning.

### 2.8 Data Analysis

Data were cleaned and screened, with outliers being identified using standardised z scores (−3 to +3), which resulted in 10 scores being winsorized (e.g., M. J. Turner et al. 2021). To address our hypotheses 7 separate one‐way ANCOVAs were conducted on each dependent variable to understand whether there were any group differences between post intervention scores. ANCOVA's were chosen as in a 2 × 2 design it decreases residual variance and produces more precise estimates, it accounts for any differences that may already exist between the groups at baseline especially as there may have been confounding variables related to those who volunteered for the experimental compared to the control group (Zhang et al. 2014). Lastly, we explored their social validation scores to understand the student's thoughts of the intervention using the principles of content analysis (Berelson 1952).

## 3 Results

### 3.1 Manipulation Checks

Students reported that they could image well (*M* = 4.61, SD = 1.19) and that they were engaged in sessions (*M* = 4.91, SD = 1.02). All descriptive statistics are presented in Table 1.

**TABLE 1:** Mean, standard deviations and effect size differences between experimental and control post variable results.

Variables	Experimental post			Control post		
	a	M	SD	M	SD	d
Stress‐mindset	0.68	3.83	0.56	3.23	0.64	1.00
Proactive coping	0.76	2.86	0.35	2.63	0.42	0.59
Performance		68.05	15.95	54.50	29.90	0.59
Irrational beliefs	0.93	92.79	14.42	84.32	13.26	0.61
Anxiety	0.84	10.29	4.03	10.64	4.66	−0.08
Depression	0.68	5.62	2.89	7.46	2.99	−0.63

*Note:* The possible ranges for Stress mindset (1–6), Irrational beliefs (1–7), Anxiety and Depression (0–3), Proactive coping (1–4), and Performance (0%–100%). *p < 0.05, **p < 0.01, ***p < 0.001

### 3.2 Stress‐Mindset

**FIGURE 1:**
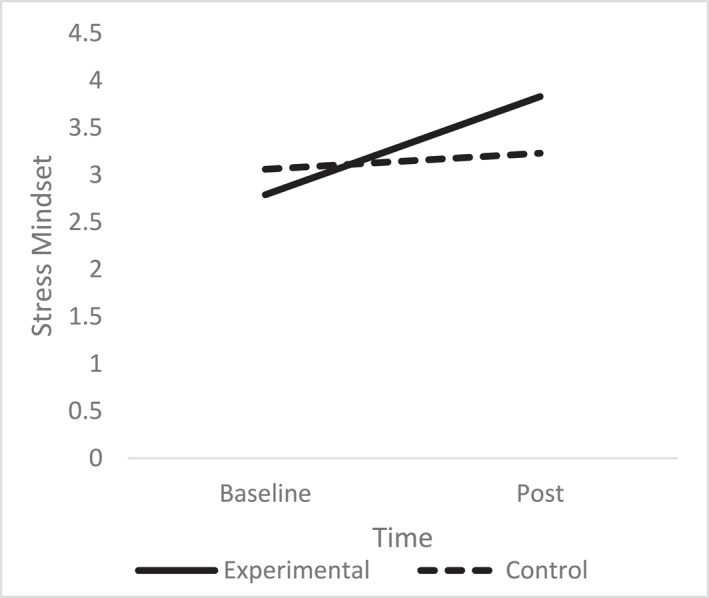
Stress mindset experimental versus control.

A one‐way ANCOVA revealed there was a significant difference in post stress mindset between the conditions (*F* (1, 68) = 12.59, *p* = < 0.001, np^2^ = 0.26), with the experimental group reporting greater stress mindset (*M* = 3.83, SD = 0.56) than the control group (*M* = 3.23, SD = 0.64), after controlling for pre intervention stress mindset scores (Figure 1).

### 3.3 Irrational Beliefs

**FIGURE 2:**
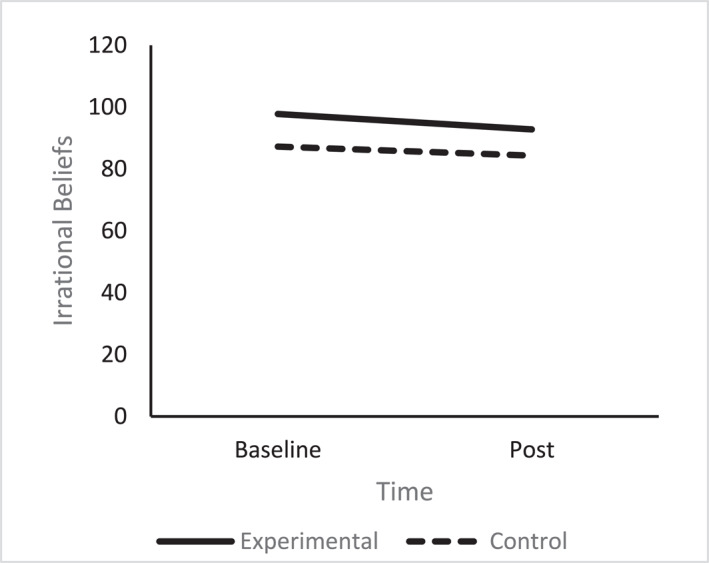
Irrational beliefs experimental versus control.

A one‐way ANCOVA revealed there was no significant difference in post irrational belief scores between the conditions (*F* (1, 68) = 3.13, *p* = 0.081, np^2^ = 0.04), after controlling for pre intervention irrational belief scores (Figure 2).

### 3.4 Proactive Coping

**FIGURE 3:**
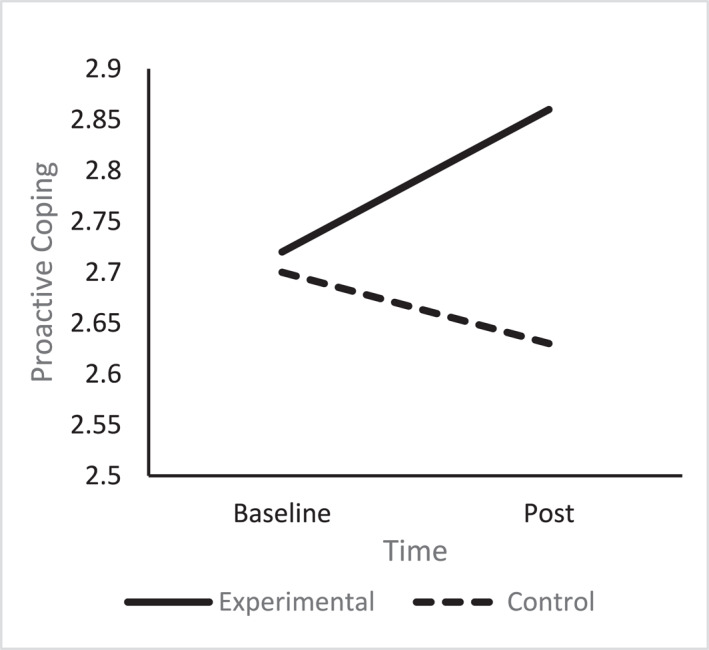
Proactive coping experimental versus control.

A one‐way ANCOVA revealed there was a significant difference in post proactive coping scores between the conditions (*F* (1, 66) = 8.63, *p* = 0.005, np^2^ = 0.12). The experimental group reported higher proactive coping scores (*M* = 2.86 SD = 0.35), compared to the control group (*M* = 2.63 SD = 0.42), see Figure 3.

### 3.5 Anxiety and Depression

**FIGURE 4:**
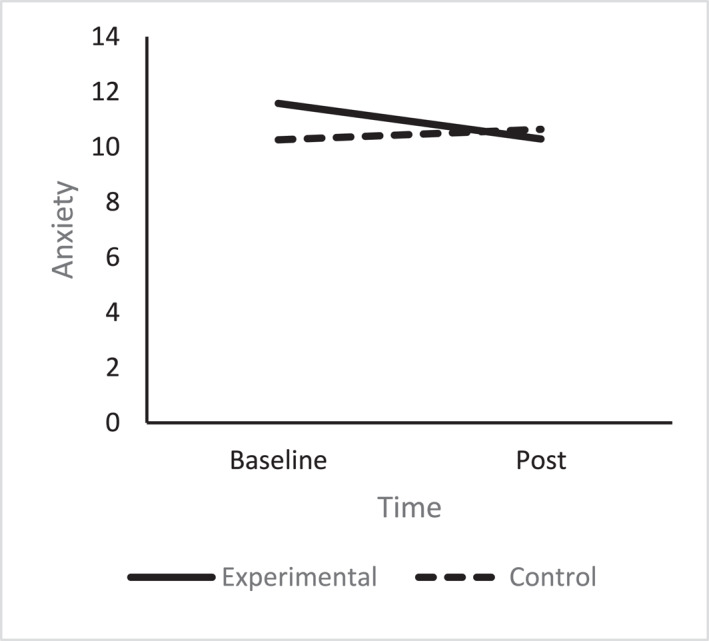
Anxiety experimental versus control.

**FIGURE 5:**
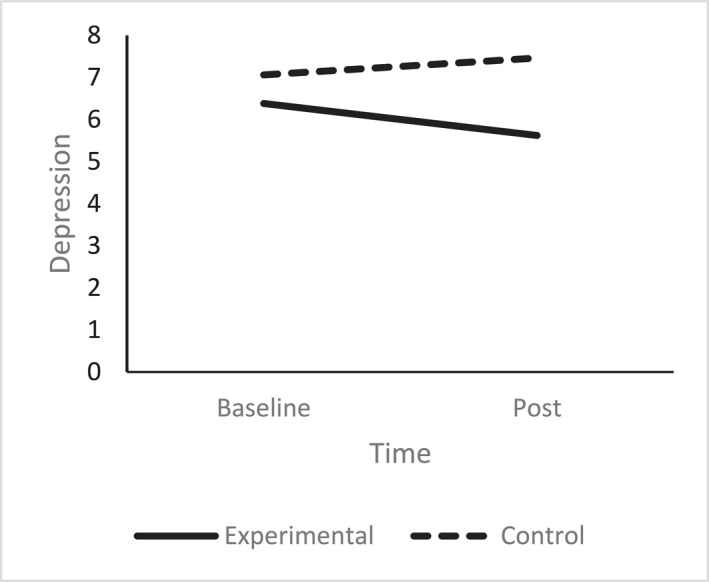
Depression experimental versus control.

A one‐way ANCOVA revealed there was no significant difference in post anxiety scores between the conditions (*F* (1, 67) = 1.85, *p* = 0.18, np^2^ = 0.03), after controlling for pre intervention anxiety scores (see Figure 4). Regarding depression, a one‐way ANCOVA revealed there was a significant difference between the conditions (*F* (1, 67) = 7.12, *p* = 0.01, np^2^ = 0.10). The experimental group reported lower depression (*M* = 5.62, SD = 2.89), compared to the control group (*M* = 7.46, SD = 2.99) after controlling for pre‐intervention scores (see Figure 5).

### 3.6 Performance

**FIGURE 6:**
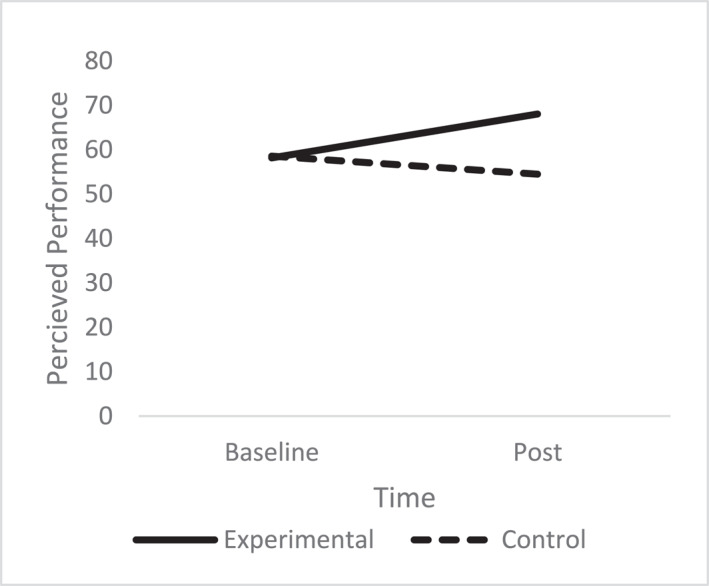
Percieved performance experimental versus control.

A one‐way ANCOVA revealed there was a significant difference in post‐performance scores between the conditions (*F* (1, 62) = 10.93, *p* = 0.002, np^2^ = 0.15). The experimental group had higher scores (*M* = 68.05%, SD = 15.95), compared to the control group (*M* = 54.50%, SD = 29.90) after controlling for pre‐intervention performance scores (See Figure 6).

## 4 Discussion

In this research we delivered a multi‐modal intervention to help adolescents facing their exams, there was improvements observed in stress mindset, proactive coping, depression and perceived performance but no changes in anxiety or irrational beliefs.

### 4.1 Hypothesis 1—Change in Stress‐Mindset

Consistent with H1 we found that students' post stress‐mindset was significantly higher in the experimental compared to the control group. Previous studies have demonstrated the malleability of stress‐mindset with this changing rapidly (Crum et al. 2017). Moreover, there was a large effect size, presenting a substantial difference in stress‐mindset following the intervention compared to the control group, similar results to the study conducted in athletes (P. Mansell et al. 2023). This may be due to the balanced content presented about stress in week one, similar results were found in Liu et al. (2019) study, whereby the adaptive short‐term benefits and detrimental effects of long‐term chronic stress were outlined.

Moreover, a balanced view of stress was reinforced throughout, specifically in session five, where participants developed their own stress mindset imagery script for their exams. Williams and Ginty (2024) found that a combination of stress mindset psychoeducation and imagery had a greater effect than the psychoeducation alone. Consistent with Lang's (1979) Bioinformational theory which proposes that imagery scripts can elicit a physiological and affective responses to a situation, it was proposed that the imagery provided an opportunity for the participants to rehearse and consolidate their new response to stress (Williams and Ginty 2024). In relation to the current study, the imagery may have provided the same opportunity for rehearsal in terms of appraising exam stress responses to be more adaptive and therefore increasing the likelihood that the new response would occur in the actual situation (Williams et al. 2017; Williams and Ginty 2024).

### 4.2 Hypothesis 2—Wellbeing Markers

In support of H2 there was a significantly higher post proactive coping scores in the experimental compared to the control, this also presented a medium‐to‐large effect size. The change in proactive coping may be due to the intervention targeting some of the antecedents of the TCTSA‐R (Meijen et al. 2020) such as the controllability. The control mapping task, for instance, whereby students reflected on what they did or did not have control over in a way that cultivated a sense of ownership on what they could influence. Additionally, in the session on self‐compassion, in realising that all humans undergo difficult situations and negative feelings, a sense of acceptance to the situation may have been promoted. In turn, this may mean that the students are more likely to feel a greater sense of controllability (Chishima et al. 2018), and therefore be proactive rather than avoidant, such as reaching out for support (K. Neff 2003; Bui et al. 2021). However, we did not measure self‐compassion, so we cannot be sure that this is the case.

Despite there being no significant difference in adolescent levels of anxiety post intervention, the experimental group revealed lower post‐depression scores compared to the control group, revealing a medium‐to‐large effect size. Our findings, therefore, support the use of multi‐model cognitive behavioural interventions for aiding mental wellbeing in adolescents approaching exams. The decrease in depression observed in the intervention group may be explained by their increase in stress mindset. Huebschmann and Sheets (2020) study, found those who reported a greater stress‐is‐enhancing mindset had lower levels of depression and anxiety under high perceived stress levels, compared to those with more of a stress‐is‐debilitating mindset. Nevertheless, lack of significant difference in anxiety may be due to the schools increasing support for mental wellbeing especially with the impending exam season, in line with the ‘Every Child Matters’ (Department for Education 2004) and ‘Mental Health and Behaviour in Schools’ (Department for Education 2018) agenda. Therefore, this may have minimised any of the significant changes that our intervention had on this variable compared to the control group, the scores are comparable between the groups.

Moreover, we measured general anxiety levels rather than test anxiety, our intervention focused mainly on strategies related to examinations, therefore may have addressed test anxiety but not general. Although general anxiety and test anxiety are connected, their relationship is complex. Individuals with high general anxiety likely to have comparable test anxiety, however those with low levels of general anxiety can still exhibit high test anxiety (Putwain 2008). Therefore, reducing test anxiety will not necessary be captured using a general anxiety measure (Putwain 2008). Furthermore, if general anxiety is comparable to test anxiety, following the intervention students may be experiencing the same level of anxiety but interpretating it as more facilitative than debilitative (Strack and Esteves 2015). Strack and Esteves (2015) found students who interpreted their anxiety more facilitative, appraised their upcoming exam as a challenge and achieved a better exam grade compared to those who interpreted their anxiety as debilitative, therefore appraising their upcoming exam as a threat. Consequently, future research should measure test anxiety and the direction.

In contrast to the H2, there was no significant difference in post irrational beliefs between the experimental and control group. This is consistent with P. Mansell et al. (2023) findings which may be due to our intervention being geared around irrational beliefs related to exams (e.g., stress and outcome), however, we used a generic irrational beliefs scale which may have not measured the change in context‐specific beliefs (P. Mansell et al. 2023). Ellis (1994) previously stated context‐specific beliefs are stronger indicators of consequences than general or nonspecific beliefs, consequently a more exam specific scale may have captured the potential change in their actual beliefs related to exams. Moreover, towards the end of the intervention examinations were more proximal, therefore it is natural for irrational beliefs become more prominent as the stressful event draws closer (Vîslă et al. 2016). This has been reported in other studies, Chadha et al. (2024), examined the temporal patterns of cognitive appraisals, irrational beliefs and challenge and threat evaluations, they found that irrational beliefs increased from 1 week before the golf tournament to the night before and 1 hour prior. Despite this being within an athletic population, similar results have been found in students. M. J. Turner et al. (2024) in a two‐wave longitudinal design found undergraduate students irrational beliefs increased from time‐point one, which was near the beginning of the academic year (October) and time‐point two, towards the main assessment period (April). Although, the current study in showing a trend towards irrational beliefs decreasing especially within the experimental group, this may be due to the intervention attenuating that possible increase in irrational beliefs that has been previously reported as a stressful event draws closer. M. J. Turner et al. (2024) emphasised that our cognitive appraisals, behaviour and beliefs may change symbolically therefore as there is a reported change in stress appraisals (cognitive appraisals) and proactive coping (behaviour), it may be argued that irrational beliefs could be improving in the same way but as beliefs are deeply rooted may take longer to change. A recent systematic review of the nature and efficacy of REBT interventions found that those that were 4 weeks or more, had greater success in changing irrational beliefs in the student population than those that were less (King et al. 2024). Consequently, the current study only explicitly focused on the framework across 2 weeks and although embedded it implicitly throughout, there may have not been enough explicit content related to REBT such as identifying the different types of rational and irrational beliefs, therefore reducing the interventions effect on their general beliefs.

### 4.3 Hypothesis 3—Performance

In support of H3, post perceived performance was significantly higher in the experimental compared to the control group, with a medium‐to‐large effect size. This positive change in the intervention group could be in part explained by the student's altered stress‐mindset (Keech, Hagger, et al. 2021; Wang et al. 2022), believing they could now harness their stress response beneficially for exams may have increased their perceived resources to meet the demands of the exams (Meijen et al. 2020; Wang et al. 2022). This is consistent with Wang et al. ’s (2022) findings, students with a stress‐is‐enhancing mindset exhibited greater challenge appraisals in anticipation to an exam, and in turn a better exam performance. In comparison those with more a stress‐is‐debilitating mindsets exhibited greater threat appraisals towards exams and a worse exam performance. Additionally, this is consistent with the positive changes witnessed in proactive coping, therefore students may have decided to start preparing for their examinations earlier through engaging with teachers support and revision, increasing their resources to face the demands.

### 4.4 Applied Implications

Teachers, pastoral staff, and parents may incorporate some of the psychoeducation of our intervention when discussing stress and demanding situations by providing their students or children with more of a balanced view of stress rather than fuelling their already pertinent and illogical belief that all stress is bad especially for exams (Crum et al. 2013). This implication is heightened by our results from the social validation with participants stating how activities such as ABC thinking, control mapping, and the self‐compassion exercise were particularly influential.

Additionally, staff members within pastoral care and teachers when exams are upcoming, may expand their repertoire of techniques away from just the typical ‘stress‐busting strategies’ that tend to be implemented with the school curriculum (Mackenzie and Williams 2018) and include some of the tools that have been utilised within this intervention. For instance, instead of relaxation strategies they may include ‘being your own best team‐mate’ (Mosewich et al. 2013) to facilitate the student's perception of resources and controllability ready for the challenging situation a head. Being equipped with many different strategies as a practitioner can help provide an individual with the best suited strategy, taking an eclectic approach (Collins and Winter 2020).

### 4.5 Limitations and Future Directions

The current study encompasses several strengths such as the successful implementation of a novel multi‐modal intervention within schools ahead of exams increasing the external validity of the results, fostered in vivo learning and allow opportunities to implement strategies in‐house. Furthermore, the intervention was underpinned by strong theoretical frameworks (e.g., REBT). Nevertheless, there are several limitations that need to be considered. First, there were unequal groups which could affect the accuracy of result comparisons made between groups and the generalisation of our findings. However, we achieved sufficient power, and this is not unusual to the intervention‐based literature, especially within the school‐based setting (Chodkiewicz and Boyle 2017). Future research could implement a wait‐list control, whereby the control group is provided the intervention following the 6‐weeks given to the experimental group. This way the control group would glean the benefits of the intervention, too. Nevertheless, there is minimal time for extra‐curricular activities within the school day and difficulties with finding sufficient physical space (Mishna et al. 2012), therefore a wait‐list control may be too challenging. Second, we did not measure self‐compassion therefore we cannot ascertain whether this was cultivated and directly played an influence on wellbeing and performance. We chose to not include this measure due to minimising the length of the questionnaire pack, reducing participant fatigue. Furthermore, the self‐compassion strategies were included to increase unconditional acceptance, consistent with REBT assumptions (Ellis 1994), therefore we assumed the result of these strategies may be captured within the measure of irrational beliefs. Third, we do not know the long‐term effects of the intervention, and, in the future, it would be beneficial to include a follow‐up measure to determine whether any changes remain temporally after the intervention.

Future studies may also consider implementing a whole‐school approach whereby the intervention is not just taught within the classroom but integrated into the school environment (Goldberg et al. 2019). If it is implemented into daily school practice and the school's culture and staff exhibit the values and principles of the intervention, reinforcement of the skills may be achieved through posters in corridors, language used by staff, topics of discussion in form times used to reiterate themes of the intervention (Goldberg et al. 2019). For instance, if the staff encompassed a stress‐is‐facilitating mindset and the school culture also espoused that, this could lead to a greater influence on a students' stress‐mindset.

## 5 Conclusion

We investigated the effect of a multi‐modal 6‐week intervention on secondary school students' mindsets, performance, and wellbeing. We found that there were significantly higher post scores of stress‐mindsets, perceived performance and proactive coping, and lower depression scores in the experimental compared to the control group, suggesting the beneficial effect of the intervention in preparing student for their exams. Contrary to our expectations there was no significant differences in post anxiety or irrational beliefs between the experimental and control group. This may be due to the intervention finishing in closer proximity of the examinations (M. J. Turner et al. 2024); however, the intervention may have acted as a buffer to some of possible increases in each of these variables. Furthermore, this is one of the first studies to implement a multimodal approach using REBT, stress mindset and self‐compassion ahead of examinations within schools in the United Kingdom. Despite several limitations such as unequal groups, no‐follow up and no measurement of self‐compassion, this study broadly supports the use of this type of intervention to help adolescents manage the demands of upcoming exams.

## Conflicts of Interest

The authors declare no conflicts of interest.

## Supporting information

Supporting Information S1

## Data Availability

The data that support the findings of this study are available from the corresponding author upon reasonable request. (Templates available at: https://authorservices.wiley.com/author‐resources/Journal‐Authors/open‐access/data‐sharing‐citation/data‐sharing‐policy.html).
