# Supplementary material for: The Influence of a Multimodal Cognitive Behavioural Intervention on the Stress Mindset, Psychological Wellbeing, and Performance of Students Aged 16–18 Facing Exams
Source: Stress Health. 2025 Jul 14;41(4):e70075. doi: 10.1002/smi.70075 (PMC12258111; doi:10.1002/smi.70075)
Supplement: Supplementary file 1 — Supporting Information S1 [file SMI-41-e70075-s001.docx]

**Appendix 1**

| Session | Tool | Aim | Content |
| --- | --- | --- | --- |
| 1 | Stress Mindset | Understand and facilitate a stress-is-enhancing mindset | - Outline of the intervention for the next 6 weeks - Discussion on the malleability of mindsets - Using a mind map students shared what they related to the word stress to find their current views - They then identified on the stress mindset continuum from stress can be helpful to stress can be unhelpful where they currently were - Students then watched a 3-minute video covering the psychoeducation of the psychological and physiological benefits of stress for performance, similar had been used in Keech et al., (2021) study. They were asked to note their three take-aways from video to maintain engagement - Group discussion was initiated on whether stress could be helpful for their exams   **Homework:** Explain the stress mindset to a friend, family member or teacher |
| 2 | Stress mindset and cognitive appraisals | Employing the stress mindset in the moment  Challenge & Threat Appraisals | - Recap of last week and reflections on homework - Using the three-step approach of acknowledge, welcome and utilise stress, produced by Crum et al. (2013), participants were taught to recognise their stress responses to be able to implement the stress mindset theory when faced with their exams - Students were introduced to Challenge and Threat and the link with stress mindset - Students were provided a task to increase their self-efficacy and controllability, called the control mapping task (Turner…. Students decided what factors could affect their exam performance. They determined what they could control (e.g. revision), influence (e.g. sleep) and what was not under their control (e.g. questions on the exam paper). Fostering greater control over the factors they could influence and understanding the factors they needed to park (uncontrollable)   **Homework:** Have a go at the control mapping task for a different pressured event e.g. sporting competition, drama show, presentation |
| 3 | A-B-C thinking | Introduced to A-B-C thinking  Facilitate rational over irrational beliefs ahead of stress & exams | - Recap of last week and reflections on homework - A short 3-minute video played that introduced the ABC framework within REBT. Students were asked to write down three take-aways from the video and share these with the cohort. - They were then provided a table of exam-related irrational and rational beliefs and students were asked to discuss which side would be more helpful. Following this they were provided an opportunity to write down their own and whether they were helpful / unhelpful. If unhelpful they had to try and switch it to the opposite. - To help facilitate a more helpful way of thinking the ‘Badness Scale’ was taught to foster perspective thinking to events that might occur (Mansell et al., 2023). A set of events were provided including ‘failing their exam’ along with ‘stubbing your toe’ and ‘losing a loved one’ etc. Students then positioned these on a scale of 0% to 100% bad. It was hoped that in this group task, students could learn to recognise and dispute their unhelpful beliefs, especially around ‘failing their exam’.   **Homework:** Outline 3-5 more helpful belief statements you could use in relation to your exam. |
| 4 | Self-Compassion | Self-compassion as a disputation technique | - Recap last week and reflections on homework - Discuss A-B-C thinking and how self-compassionate could dispute unhelpful beliefs - Students learnt the difference between self-critical and self-compassionate thoughts but also to acknowledge, share and show self-kindness towards their exams. - The practical task asked students to consider how they might think and feel before an exam and after a bad result. By being their own ‘support coach’, they were then asked to write how they might advise themselves on how to respond to such thoughts and feelings (e.g. changing a thought from ‘I am a failure’ to ‘I didn’t do as well as I thought I would, but I tried my best, we’re all fallible human beings’. The task was also encouraged common humanity, as when individuals shared their thoughts and feelings, it was clear everyone thought similarly towards exams. Consequently, this encouraged reaching out for support, as it normalised feelings of anxiety or worry over failure.   **Homework:** Practicing the support coach strategy when self-criticism arises |
| 5 | Imagery | How to use imagery and develop their own script | - Recap last week and reflections on homework - Introduction to what imagery is and why it is a helpful skill - Students then experienced imagery through the ‘Tangy Lemon’ task (Keech et al., 2021). - Explanation on how imagery can be used to reappraise stress responses, following this they experienced an imagery script for themselves which was based around exam and the three-step approach as outlined in week 2. - Students were then guided through the formulation of an imagery script which aimed to integrate stress mindset theory under headings of ‘acknowledge’, ‘welcome’ and ‘utilise’ (e.g., Crum et al., 2013). To illustrate, an example from a student was “As I approach the exam hall, I feel my heart racing, but this is just one of the ways that my body is preparing me to focus”. The co-creation of a script enabled the personalisation of the script within a theoretical framework, resulting in it being more realistic and meaningful (Quinton et al., 2019).   **Homework:** To record their script and listen back to it |
| 6 | Recap | Recap and reiterate | - Recep last week and reflections on homework - Revisited each of the key psychoeducation and strategies from the intervention, and to offer ways in which participants could embed the strategies into their daily lives. |
